# *c*-3,*t*-3-Dimethyl-*r*-2,*c*-7-diphenyl-1,4-diazepan-5-one

**DOI:** 10.1107/S160053680904330X

**Published:** 2009-10-28

**Authors:** K. Ravichandran, P. Ramesh, S. Sethuvasan, S. Ponnuswamy, M. N. Ponnuswamy

**Affiliations:** a Centre of Advanced Study in Crystallography and Biophysics, University of Madras, Guindy Campus, Chennai 600 025, India; b Department of Chemistry, Government Arts College (Autonomous), Coimbatore 641 018, India

## Abstract

In the title compound, C_19_H_22_N_2_O, the diazepine ring adopts a distorted chair conformation. One of the N—H groups forms an inter­molecular N—H⋯O hydrogen bond generating an *R*_2_^2^(8) graph-set motif. The other N—H group does not form a hydrogen bond.

## Related literature

For general background to diazepine derivatives, see: Hirokawa *et al.* (1998 ▶); Jeyaraman & Ponnuswamy (1997 ▶). For asymmetry parameters, see: Nardelli (1983 ▶). For puckering parameters, see: Cremer & Pople (1975 ▶). For hydrogen-bond motifs, see: Bernstein *et al.* (1995 ▶). For the synthesis, see: Jeyaraman *et al.* (1995 ▶); Ponnuswamy *et al.* (2006 ▶).
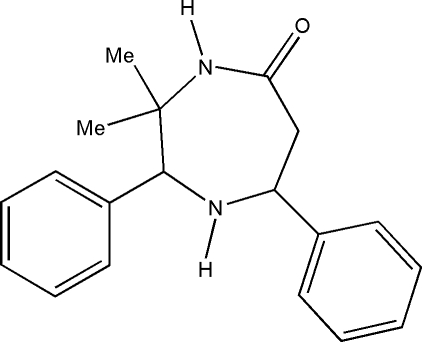


## Experimental

### 

#### Crystal data

C_19_H_22_N_2_O
*M* *_r_* = 294.39
Triclinic, 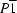

*a* = 6.7354 (4) Å
*b* = 10.6867 (6) Å
*c* = 11.4186 (7) Å
α = 82.191 (3)°
β = 88.218 (4)°
γ = 80.317 (3)°
*V* = 802.65 (8) Å^3^
*Z* = 2
Mo *K*α radiation
μ = 0.08 mm^−1^
*T* = 293 K
0.25 × 0.20 × 0.20 mm

#### Data collection

Bruker Kappa APEXII area-detector diffractometer
Absorption correction: multi-scan (*SADABS*; Sheldrick, 2001 ▶) *T*_min_ = 0.982, *T*_max_ = 0.985
17703 measured reflections
3958 independent reflections
3196 reflections with *I* > 2σ(*I*)
*R*_int_ = 0.029

#### Refinement

*R*[*F* ^2^ > 2σ(*F* ^2^)] = 0.061
*wR*(*F* ^2^) = 0.167
*S* = 1.08
3958 reflections
209 parameters
H atoms treated by a mixture of independent and constrained refinement
Δρ_max_ = 0.26 e Å^−3^
Δρ_min_ = −0.23 e Å^−3^

### 

Data collection: *APEX2* (Bruker, 2004 ▶); cell refinement: *SAINT* (Bruker, 2004 ▶); data reduction: *SAINT*; program(s) used to solve structure: *SHELXS97* (Sheldrick, 2008 ▶); program(s) used to refine structure: *SHELXL97* (Sheldrick, 2008 ▶); molecular graphics: *ORTEP-3* (Farrugia, 1997 ▶); software used to prepare material for publication: *SHELXL97* and *PLATON* (Spek, 2009 ▶).

## Supplementary Material

Crystal structure: contains datablocks global, I. DOI: 10.1107/S160053680904330X/bt5093sup1.cif

Structure factors: contains datablocks I. DOI: 10.1107/S160053680904330X/bt5093Isup2.hkl

Additional supplementary materials:  crystallographic information; 3D view; checkCIF report

## Figures and Tables

**Table 1:** Hydrogen-bond geometry (Å, °)

*D*—H⋯*A*	*D*—H	H⋯*A*	*D*⋯*A*	*D*—H⋯*A*
N1—H1⋯O1^i^	0.90 (3)	2.02 (3)	2.928 (2)	177 (2)

Symmetry code: (i) 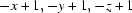.

**Fig. 1.**
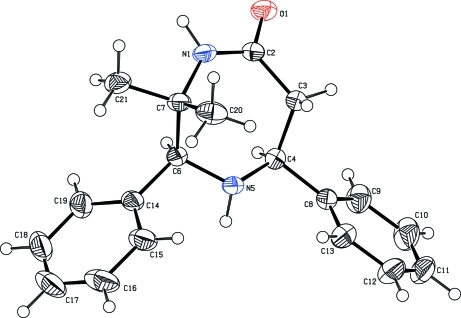
Perspective view of the molecule showing the displacement ellipsoids at the 30% probability level. H atoms have been omitted for clarity.

## supplementary crystallographic information

### Comment

1,4-Diazepines are of considerable importance due to their wide spectrum of biological activities (Hirokawa *et al.*, 1998). Various substituted diazepin-5-ones have been synthesized using Schmidt rearrangement from the corresponding piperdin-4-ones and their stereochemistry has been reported (Jeyaraman & Ponnuswamy, 1997). In view of these importance and to ascertain the molecular conformation, crystallographic study of the title compound, namely *c*-3,*t*-3-dimethyl-*r*-2,*c*-7-diphenyl-1,4-diazepan-5-one, has been carried out.

The *ORTEP* diagram of the title compound is shown in Fig. 1. The diazepine ring adopts a distorted chair conformation with puckering parameters (Cremer & Pople, 1975) and asymmetry parameters (Nardelli, 1983) of q_2_ = 0.348 (2)Å, q_3_ = 0.677 (2)Å, φ_2_ = 105.2 (3)°, φ_3_ =99.9 (2)° and Δ_s_(N5)= 12.2 (2)°. The sum of the bond angles around the N1 atom (359.4°) of the diazepine ring is in *sp*^2^-hybridization, whereas the other atom, N5 (331.1°), is in accordance with *sp*^3^-hybridization.

The crystal packing is stabilized by intermolecular N—H···O interactions. The molecules at (*x*, *y*, *z*) and (-*x*+1, -*y*+1, -*z*+1) are linked through intermolecular N1—H1···O1 hydrogen bonds into cyclic centrosymmetric *R*_2_^2^(8) dimers (Bernstein *et al.* 1995).

### Experimental

In a typical reaction, c-3,t-3-dimethyl-r-2,c-6-diphenylpiperidin-4-one was first converted into its hydrochloride and the dry, powdered c-3,t-3-dimethyl-r-2,c-6-diphenylpiperidin-4-one hydrochloride (10.0 g) was added, in portions, to cold conc. H_2_SO_4_ (25.0 ml). The temperature of the solution was allowed to rise to 25°C and NaN_3_ (3.0 g) was added in portions with vigorous stirring. The solution was poured into crushed ice and cold NaOH solution (2 N) was added slowly with stirring until the pH was 8. The separated white solid was filtered and crystallized using ethanol and pet-ether (60–80°C) in the ratio of 9.5:0.5 (Jeyaraman *et al.*, 1995; Ponnuswamy *et al.*, 2006).

### Refinement

The amino H atoms were refined and the other H atoms positioned geometrically (C—H=0.93–0.98 Å) and allowed to ride on their parent atoms, with 1.5*U*_eq_(C) for methyl H and 1.2 *U*_eq_(C) for other H atoms.

### Crystal data

**Table:**

C_19_H_22_N_2_O	*Z* = 2
*M**_r_* = 294.39	*F*(000) = 316
Triclinic, *P*1	*D*_x_ = 1.218 Mg m^−^^3^
Hall symbol: -P 1	Mo *K*α radiation, λ = 0.71073 Å
*a* = 6.7354 (4) Å	Cell parameters from 3562 reflections
*b* = 10.6867 (6) Å	θ = 2.5–28.4°
*c* = 11.4186 (7) Å	µ = 0.08 mm^−^^1^
α = 82.191 (3)°	*T* = 293 K
β = 88.218 (4)°	Block, colorless
γ = 80.317 (3)°	0.25 × 0.20 × 0.20 mm
*V* = 802.65 (8) Å^3^	

### Data collection

**Table:**

Bruker Kappa APEXII area-detector diffractometer	3958 independent reflections
Radiation source: fine-focus sealed tube	3196 reflections with *I* > 2σ(*I*)
graphite	*R*_int_ = 0.029
ω and φ scans	θ_max_ = 28.4°, θ_min_ = 2.5°
Absorption correction: multi-scan (*SADABS*; Sheldrick, 2001)	*h* = −8→8
*T*_min_ = 0.982, *T*_max_ = 0.985	*k* = −14→14
17703 measured reflections	*l* = −15→14

### Refinement

**Table:**

Refinement on *F*^2^	Primary atom site location: structure-invariant direct methods
Least-squares matrix: full	Secondary atom site location: difference Fourier map
*R*[*F*^2^ > 2σ(*F*^2^)] = 0.061	Hydrogen site location: inferred from neighbouring sites
*wR*(*F*^2^) = 0.167	H atoms treated by a mixture of independent and constrained refinement
*S* = 1.08	*w* = 1/[σ^2^(*F*_o_^2^) + (0.0617*P*)^2^ + 0.472*P*] where *P* = (*F*_o_^2^ + 2*F*_c_^2^)/3
3958 reflections	(Δ/σ)_max_ < 0.001
209 parameters	Δρ_max_ = 0.26 e Å^−^^3^
0 restraints	Δρ_min_ = −0.23 e Å^−^^3^

### Special details

**Table:**

Geometry. All e.s.d.'s (except the e.s.d. in the dihedral angle between two l.s. planes) are estimated using the full covariance matrix. The cell e.s.d.'s are taken into account individually in the estimation of e.s.d.'s in distances, angles and torsion angles; correlations between e.s.d.'s in cell parameters are only used when they are defined by crystal symmetry. An approximate (isotropic) treatment of cell e.s.d.'s is used for estimating e.s.d.'s involving l.s. planes.
Refinement. Refinement of *F*^2^ against ALL reflections. The weighted *R*-factor *wR* and goodness of fit *S* are based on *F*^2^, conventional *R*-factors *R* are based on *F*, with *F* set to zero for negative *F*^2^. The threshold expression of *F*^2^ > σ(*F*^2^) is used only for calculating *R*-factors(gt) *etc*. and is not relevant to the choice of reflections for refinement. *R*-factors based on *F*^2^ are statistically about twice as large as those based on *F*, and *R*- factors based on ALL data will be even larger.

### Fractional atomic coordinates and isotropic or equivalent isotropic displacement parameters (Å^2^)

**Table:**

	*x*	*y*	*z*	*U*_iso_*/*U*_eq_	
O1	0.3091 (2)	0.63384 (13)	0.48222 (13)	0.0459 (4)	
N1	0.3506 (2)	0.48085 (15)	0.36366 (15)	0.0377 (4)	
H1	0.453 (4)	0.443 (2)	0.412 (2)	0.053 (6)*	
C2	0.2588 (3)	0.59310 (17)	0.39357 (16)	0.0350 (4)	
C3	0.0919 (3)	0.67316 (17)	0.31721 (18)	0.0390 (4)	
H3A	0.1428	0.6879	0.2370	0.047*	
H3B	0.0573	0.7558	0.3453	0.047*	
C4	−0.0997 (3)	0.61476 (16)	0.31459 (16)	0.0332 (4)	
H4	−0.1374	0.5842	0.3957	0.040*	
N5	−0.0711 (2)	0.50751 (14)	0.24522 (14)	0.0358 (4)	
H5	−0.191 (3)	0.4839 (19)	0.2375 (18)	0.037 (5)*	
C6	0.0652 (2)	0.39247 (15)	0.29509 (16)	0.0313 (4)	
H6	0.0410	0.3803	0.3806	0.038*	
C7	0.2915 (3)	0.40542 (17)	0.27481 (16)	0.0343 (4)	
C8	−0.2685 (3)	0.71712 (16)	0.26079 (17)	0.0350 (4)	
C9	−0.3855 (3)	0.7958 (2)	0.3313 (2)	0.0503 (5)	
H9	−0.3629	0.7842	0.4123	0.060*	
C10	−0.5362 (4)	0.8919 (2)	0.2834 (3)	0.0650 (7)	
H10	−0.6130	0.9447	0.3322	0.078*	
C11	−0.5727 (3)	0.9095 (2)	0.1653 (3)	0.0652 (7)	
H11	−0.6747	0.9736	0.1333	0.078*	
C12	−0.4587 (4)	0.8324 (2)	0.0943 (3)	0.0635 (7)	
H12	−0.4828	0.8445	0.0135	0.076*	
C13	−0.3075 (3)	0.7363 (2)	0.1414 (2)	0.0482 (5)	
H13	−0.2314	0.6840	0.0919	0.058*	
C14	0.0070 (3)	0.27925 (17)	0.24575 (18)	0.0370 (4)	
C15	−0.0316 (3)	0.2835 (2)	0.1270 (2)	0.0499 (5)	
H15	−0.0186	0.3566	0.0750	0.060*	
C16	−0.0896 (4)	0.1794 (3)	0.0848 (3)	0.0680 (8)	
H16	−0.1138	0.1828	0.0046	0.082*	
C17	−0.1115 (4)	0.0715 (3)	0.1608 (3)	0.0749 (9)	
H17	−0.1501	0.0017	0.1325	0.090*	
C18	−0.0763 (4)	0.0676 (2)	0.2778 (3)	0.0692 (8)	
H18	−0.0918	−0.0053	0.3296	0.083*	
C19	−0.0175 (3)	0.17043 (19)	0.3214 (2)	0.0510 (5)	
H19	0.0055	0.1662	0.4018	0.061*	
C20	0.3380 (3)	0.4639 (2)	0.14928 (18)	0.0493 (5)	
H20A	0.2536	0.5458	0.1310	0.074*	
H20B	0.3127	0.4082	0.0943	0.074*	
H20C	0.4768	0.4746	0.1437	0.074*	
C21	0.4225 (3)	0.2742 (2)	0.3012 (2)	0.0470 (5)	
H21A	0.5614	0.2841	0.3029	0.071*	
H21B	0.4042	0.2230	0.2407	0.071*	
H21C	0.3845	0.2328	0.3764	0.071*	

### Atomic displacement parameters (Å^2^)

**Table:**

	*U*^11^	*U*^22^	*U*^33^	*U*^12^	*U*^13^	*U*^23^
O1	0.0448 (8)	0.0471 (8)	0.0484 (8)	−0.0047 (6)	−0.0165 (6)	−0.0155 (6)
N1	0.0325 (8)	0.0402 (8)	0.0414 (9)	−0.0027 (6)	−0.0140 (7)	−0.0102 (6)
C2	0.0321 (9)	0.0366 (9)	0.0381 (9)	−0.0098 (7)	−0.0081 (7)	−0.0045 (7)
C3	0.0395 (10)	0.0304 (8)	0.0481 (11)	−0.0066 (7)	−0.0146 (8)	−0.0044 (7)
C4	0.0335 (9)	0.0301 (8)	0.0354 (9)	−0.0036 (6)	−0.0043 (7)	−0.0032 (6)
N5	0.0279 (7)	0.0329 (7)	0.0481 (9)	−0.0036 (6)	−0.0112 (6)	−0.0098 (6)
C6	0.0273 (8)	0.0301 (8)	0.0368 (9)	−0.0034 (6)	−0.0037 (6)	−0.0060 (6)
C7	0.0279 (8)	0.0397 (9)	0.0376 (9)	−0.0056 (7)	−0.0060 (7)	−0.0113 (7)
C8	0.0292 (8)	0.0309 (8)	0.0446 (10)	−0.0061 (6)	−0.0036 (7)	−0.0022 (7)
C9	0.0463 (12)	0.0448 (11)	0.0570 (13)	−0.0004 (9)	0.0059 (10)	−0.0072 (9)
C10	0.0439 (12)	0.0467 (12)	0.099 (2)	0.0065 (10)	0.0125 (13)	−0.0097 (13)
C11	0.0375 (12)	0.0496 (12)	0.100 (2)	0.0004 (9)	−0.0148 (12)	0.0137 (13)
C12	0.0550 (14)	0.0604 (14)	0.0699 (16)	−0.0067 (11)	−0.0253 (12)	0.0121 (12)
C13	0.0457 (11)	0.0468 (11)	0.0497 (12)	−0.0016 (9)	−0.0111 (9)	−0.0032 (9)
C14	0.0239 (8)	0.0342 (8)	0.0545 (11)	−0.0040 (6)	−0.0018 (7)	−0.0125 (8)
C15	0.0437 (11)	0.0544 (12)	0.0573 (13)	−0.0126 (9)	−0.0076 (10)	−0.0202 (10)
C16	0.0493 (13)	0.0785 (18)	0.0888 (19)	−0.0147 (12)	−0.0088 (13)	−0.0494 (16)
C17	0.0434 (13)	0.0535 (14)	0.141 (3)	−0.0130 (10)	−0.0009 (15)	−0.0523 (17)
C18	0.0470 (13)	0.0324 (10)	0.129 (3)	−0.0067 (9)	−0.0004 (15)	−0.0145 (13)
C19	0.0399 (11)	0.0357 (10)	0.0763 (16)	−0.0042 (8)	−0.0006 (10)	−0.0068 (9)
C20	0.0415 (11)	0.0707 (14)	0.0408 (11)	−0.0208 (10)	0.0030 (8)	−0.0116 (10)
C21	0.0320 (10)	0.0484 (11)	0.0615 (13)	0.0035 (8)	−0.0095 (9)	−0.0205 (9)

### Geometric parameters (Å, °)

**Table:**

O1—C2	1.233 (2)	C10—H10	0.9300
N1—C2	1.337 (2)	C11—C12	1.362 (4)
N1—C7	1.481 (2)	C11—H11	0.9300
N1—H1	0.90 (3)	C12—C13	1.383 (3)
C2—C3	1.513 (2)	C12—H12	0.9300
C3—C4	1.528 (2)	C13—H13	0.9300
C3—H3A	0.9700	C14—C19	1.381 (3)
C3—H3B	0.9700	C14—C15	1.382 (3)
C4—N5	1.463 (2)	C15—C16	1.388 (3)
C4—C8	1.519 (2)	C15—H15	0.9300
C4—H4	0.9800	C16—C17	1.372 (4)
N5—C6	1.463 (2)	C16—H16	0.9300
N5—H5	0.89 (2)	C17—C18	1.358 (4)
C6—C14	1.515 (2)	C17—H17	0.9300
C6—C7	1.561 (2)	C18—C19	1.385 (3)
C6—H6	0.9800	C18—H18	0.9300
C7—C21	1.523 (3)	C19—H19	0.9300
C7—C20	1.529 (3)	C20—H20A	0.9600
C8—C13	1.377 (3)	C20—H20B	0.9600
C8—C9	1.378 (3)	C20—H20C	0.9600
C9—C10	1.384 (3)	C21—H21A	0.9600
C9—H9	0.9300	C21—H21B	0.9600
C10—C11	1.360 (4)	C21—H21C	0.9600
			
C2—N1—C7	129.23 (15)	C9—C10—H10	119.8
C2—N1—H1	113.2 (15)	C10—C11—C12	119.5 (2)
C7—N1—H1	117.0 (15)	C10—C11—H11	120.2
O1—C2—N1	121.10 (16)	C12—C11—H11	120.2
O1—C2—C3	118.95 (16)	C11—C12—C13	120.6 (2)
N1—C2—C3	119.94 (16)	C11—C12—H12	119.7
C2—C3—C4	115.13 (15)	C13—C12—H12	119.7
C2—C3—H3A	108.5	C8—C13—C12	120.7 (2)
C4—C3—H3A	108.5	C8—C13—H13	119.7
C2—C3—H3B	108.5	C12—C13—H13	119.7
C4—C3—H3B	108.5	C19—C14—C15	118.53 (19)
H3A—C3—H3B	107.5	C19—C14—C6	119.65 (19)
N5—C4—C8	109.15 (14)	C15—C14—C6	121.76 (18)
N5—C4—C3	111.58 (15)	C14—C15—C16	120.5 (2)
C8—C4—C3	109.06 (14)	C14—C15—H15	119.8
N5—C4—H4	109.0	C16—C15—H15	119.8
C8—C4—H4	109.0	C17—C16—C15	120.3 (3)
C3—C4—H4	109.0	C17—C16—H16	119.8
C4—N5—C6	116.12 (14)	C15—C16—H16	119.8
C4—N5—H5	108.4 (13)	C18—C17—C16	119.4 (2)
C6—N5—H5	106.6 (13)	C18—C17—H17	120.3
N5—C6—C14	107.83 (13)	C16—C17—H17	120.3
N5—C6—C7	112.49 (14)	C17—C18—C19	121.1 (3)
C14—C6—C7	113.70 (14)	C17—C18—H18	119.5
N5—C6—H6	107.5	C19—C18—H18	119.5
C14—C6—H6	107.5	C14—C19—C18	120.2 (2)
C7—C6—H6	107.5	C14—C19—H19	119.9
N1—C7—C21	104.82 (14)	C18—C19—H19	119.9
N1—C7—C20	111.22 (16)	C7—C20—H20A	109.5
C21—C7—C20	108.80 (17)	C7—C20—H20B	109.5
N1—C7—C6	108.63 (14)	H20A—C20—H20B	109.5
C21—C7—C6	109.66 (15)	C7—C20—H20C	109.5
C20—C7—C6	113.36 (15)	H20A—C20—H20C	109.5
C13—C8—C9	117.95 (18)	H20B—C20—H20C	109.5
C13—C8—C4	121.90 (17)	C7—C21—H21A	109.5
C9—C8—C4	120.15 (18)	C7—C21—H21B	109.5
C8—C9—C10	120.9 (2)	H21A—C21—H21B	109.5
C8—C9—H9	119.5	C7—C21—H21C	109.5
C10—C9—H9	119.5	H21A—C21—H21C	109.5
C11—C10—C9	120.3 (2)	H21B—C21—H21C	109.5
C11—C10—H10	119.8		
			
C7—N1—C2—O1	168.29 (18)	C3—C4—C8—C9	−87.3 (2)
C7—N1—C2—C3	−12.5 (3)	C13—C8—C9—C10	−0.6 (3)
O1—C2—C3—C4	−114.2 (2)	C4—C8—C9—C10	178.2 (2)
N1—C2—C3—C4	66.6 (2)	C8—C9—C10—C11	0.6 (4)
C2—C3—C4—N5	−73.1 (2)	C9—C10—C11—C12	−0.5 (4)
C2—C3—C4—C8	166.24 (16)	C10—C11—C12—C13	0.4 (4)
C8—C4—N5—C6	−171.21 (15)	C9—C8—C13—C12	0.5 (3)
C3—C4—N5—C6	68.2 (2)	C4—C8—C13—C12	−178.3 (2)
C4—N5—C6—C14	155.20 (16)	C11—C12—C13—C8	−0.4 (4)
C4—N5—C6—C7	−78.64 (19)	N5—C6—C14—C19	−131.39 (18)
C2—N1—C7—C21	−166.7 (2)	C7—C6—C14—C19	103.2 (2)
C2—N1—C7—C20	75.9 (2)	N5—C6—C14—C15	45.6 (2)
C2—N1—C7—C6	−49.5 (3)	C7—C6—C14—C15	−79.8 (2)
N5—C6—C7—N1	79.63 (18)	C19—C14—C15—C16	−1.3 (3)
C14—C6—C7—N1	−157.44 (15)	C6—C14—C15—C16	−178.35 (19)
N5—C6—C7—C21	−166.35 (15)	C14—C15—C16—C17	0.7 (4)
C14—C6—C7—C21	−43.4 (2)	C15—C16—C17—C18	0.1 (4)
N5—C6—C7—C20	−44.5 (2)	C16—C17—C18—C19	−0.4 (4)
C14—C6—C7—C20	78.4 (2)	C15—C14—C19—C18	1.1 (3)
N5—C4—C8—C13	−30.7 (2)	C6—C14—C19—C18	178.18 (18)
C3—C4—C8—C13	91.4 (2)	C17—C18—C19—C14	−0.2 (3)
N5—C4—C8—C9	150.53 (18)		

### Hydrogen-bond geometry (Å, °)

**Table:**

*D*—H···*A*	*D*—H	H···*A*	*D*···*A*	*D*—H···*A*
N1—H1···O1^i^	0.90 (3)	2.02 (3)	2.928 (2)	177 (2)

Symmetry codes: (i) −*x*+1, −*y*+1, −*z*+1.
